# Management of hindfoot and ankle in Charcot arthropathy

**DOI:** 10.1530/EOR-2025-0057

**Published:** 2025-06-02

**Authors:** Nicolas Cellier

**Affiliations:** Department of Orthopedic and Traumatological Surgery, Spine surgery - Pr Kouyoumdjian. Caremeau University Hospital, Nîmes, France

**Keywords:** Charcot, foot, management, diabetes, surgical, hindfoot, ankle, neuroarthropathy

## Abstract

- Charcot neuroarthropathy is the most severe complication of the diabetic foot. Its diagnosis is difficult and often overlooked, delaying management, with sometimes disastrous consequences. Its incidence is increasing due to the rapid global rise in the number of people with diabetes.
- Its pathophysiology remains unclear, although the activation of the RANK/RANK-L system appears to be involved, triggered either by neurotraumatic or neurovascular mechanisms, leading to the differentiation of monocytes into osteoclasts.
- Diagnosis relies on clinical and radiological arguments, particularly MRI.
- There are different types of Charcot foot depending on the evolution, according to Eichenholtz’s classification and based on location according to Sanders and Brodsky’s classifications.
- Treatment involves a multidisciplinary approach with diabetes management and addressing other general complications. Medical treatment is indicated as the first line, with offloading and immobilisation using a ‘total contact cast’. In case of failure of this method, or if there is immediate deformity, surgical intervention is indicated, and techniques are evolving rapidly. Depending on the deformity, minimally invasive or arthroscopic procedures may be performed. In cases of significant deformity, foot reconstruction may be proposed, using the so-called ‘super construct’ technique if necessary. Infection will be treated concurrently or initially, depending on severity.
- Many complications are reported, but increasingly early and aggressive surgery improves patients’ quality of life and reduces amputation rates.

## Introduction

According to the International Diabetes Federation, the number of diabetic patients worldwide is expected to increase by 46% by 2045, reaching approximately 783 million people. Consequently, the incidence of Charcot neuroarthropathy is expected to follow the same increase (1). It is the ultimate complication of the diabetic foot, occurring after an average duration of diabetes exceeding 10 years, with an incidence of 6.4–9.5 per 10,000 (2). First described by Jean-Martin Charcot in 1868, it was in 1936 that William Riely Jordan linked Charcot neuroarthropathy to diabetes, which is currently its main cause (3). The pathophysiology is currently poorly understood, but it appears that the activation of the RANK/RANK-L axis, leading to the differentiation of monocytes into osteoclasts, is the primary aetiology of Charcot foot (4). Diagnosis is also challenging, leading to delayed management in 25% of cases (5), with major foot deformities potentially resulting in wounds and osteoarticular infections. In the early stage, medical treatment involving offloading and immobilisation is indicated, and no pharmacological treatment has proven effective in reducing the inflammatory phase. In more advanced stages, or in cases of unfavourable evolution of early medical treatment, surgical treatment may be proposed based on the anatomical location of Charcot foot. Techniques are evolving rapidly (6). This will involve stabilising the foot, correcting morphostatic disorders and managing osteoarticular infections if necessary (7). Various techniques have been described, but mixed osteosynthesis (internal and external) seems to yield the best results, even though the complication rate is significant, potentially leading to amputation (8).

## Epidemiology

The incidence of Charcot foot ranges from 0.3 to 0.85% in the type 2 diabetic population (9, 10). Type 1 diabetic patients are affected earlier, around their third or fourth decade, while type 2 patients are more commonly affected in their sixth or seventh decade (11). The mortality rate is 30% at 5 years, with an average reduction in life expectancy of 14 years compared to the general population. Charcot neuroarthropathy increases the risk of foot ulcers by up to 30%, and the risk of amputation is multiplied by 7–12, affecting approximately 25% of patients. Charcot foot decreases quality of life due to foot deformity, loss of mobility and thus autonomy, as well as frequent hospitalisations (9).

## Pathophysiology

The causes of Charcot neuroarthropathy and its sequelae are multifactorial. There is no Charcot foot without neuropathy. Numerous risk factors have been identified: duration of diabetes, presence of retinopathy, microalbuminuria and macroalbuminuria, elevated HbA1C levels and the presence of atherosclerosis (2). Currently, two hypotheses stand out: the neurovascular theory and the neurotraumatic theory. Regarding the former, it involves a hyperaemic state secondary to damage to the sympathetic nervous system, leading to venous hypertension that causes suffering of the soft tissues of the foot. This suffering prevents the maintenance of the foot’s architecture. This hyperaemia is also responsible for the activation of the RANK/RANK-L system, which would cause an imbalance by activating bone resorption through the differentiation of monocytes into osteoclasts (12). As for the neurotraumatic theory, it is attributed to repeated microtraumas to the lower limb, where the loss of proprioception triggers a pro-inflammatory process that activates osteoclastogenesis through the same enzyme system. The loading of the foot on these inflammatory processes leads to failure of the muscles supporting the foot, and fractures responsible for instability, leading to its collapse.

## Diagnosis

The clinical diagnosis of acute Charcot foot is challenging. According to the recommendations of the International Working Group on Diabetic Foot (IWGDF), the clinical diagnosis is based on the association, in a diabetic patient with neuropathy and without skin lesions, of local temperature elevation with oedema and redness of the foot compared to the contralateral foot (13). Temperature should be measured using an infrared thermometer at the ankle and foot at the same location on each side (14). The paraclinical assessment relies on a weight-bearing, bilateral and comparative radiographic evaluation (15). In the case of normal radiographic findings, MRI is recommended to confirm or rule out the diagnosis and quantify its activity if necessary. If MRI is contraindicated, CT, scintigraphy or PET scans may be performed (16). It is not recommended to conduct blood tests such as C-reactive protein (CRP) or erythrocyte sedimentation rate (ESR), as no biological marker allows for early diagnosis (17).

## Classification

**Figure 1:**
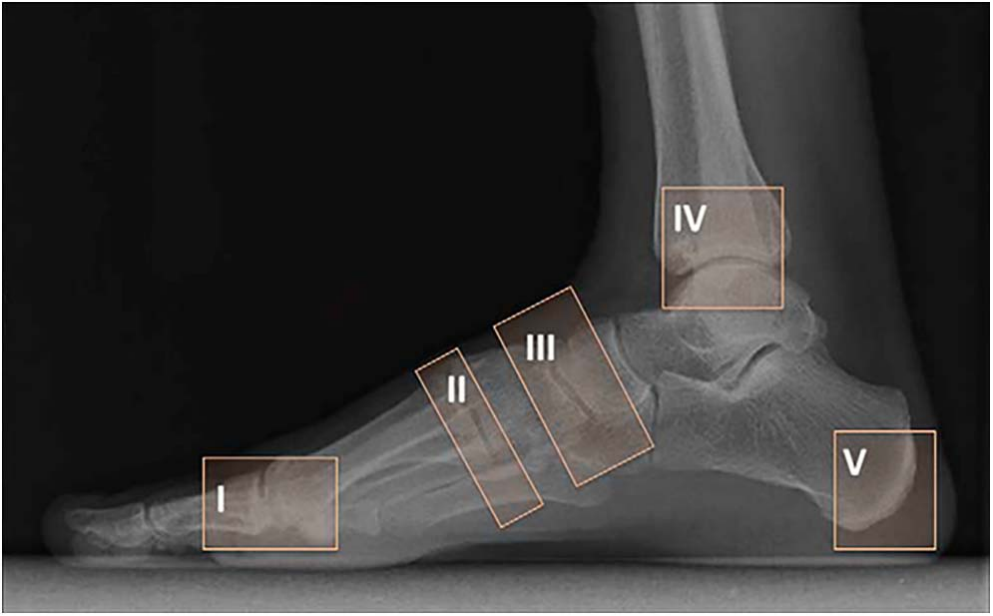
Sanders classification.

**Figure 2:**
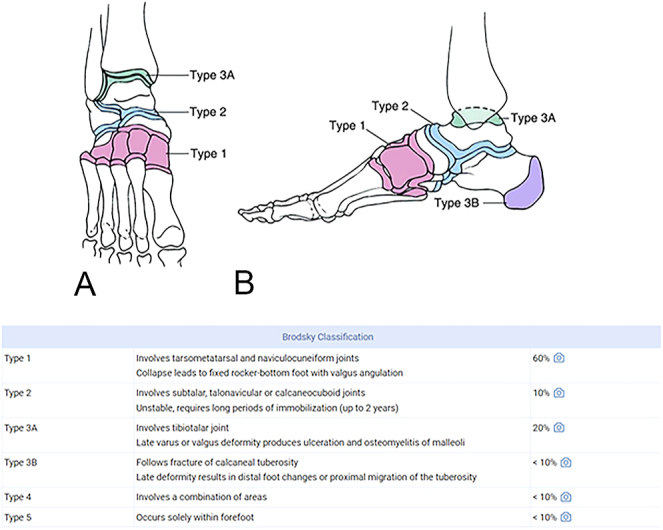
Brodsky classification.

Several classifications facilitate the analysis of Charcot foot. Eichenholtz’s classification evaluates the temporal evolution of the disease, while Sanders’ and, more recently, Brodsky’s classifications analyse the anatomical location (18). The modified Eichenholtz classification is summarised in Table 1 (19). This historical classification is now supplanted by the MRI classification into four stages as well (Table 2) (15), although Eichenholtz’s classification remains widely used. Regarding the anatomical level of Charcot neuroarthropathy involvement, Sanders’ classification (Fig. 1), historically simple, and more recently Brodsky’s classification (Fig. 2) are utilised (20). These classifications are based on the most frequently affected anatomical regions. Brodsky’s classification has the advantage of considering hindfoot dislocations. The midfoot and hindfoot are the most commonly affected locations.

**Table 1:** Eichenholtz classification.

Stage	Radiographic findings	Clinical findings	Treatment
0 (prodromal)	Normal radiographs	Swelling, erythema, warmth	Patient education, serial radiographs to monitor progression, protected weight-bearing
I (development)	Osteopenia, fragmentation, joint subluxation or dislocation	Swelling, erythema, warmth, ligamentous laxity	Protected weight-bearing with total contact casting or prefabricated pneumatic brace. Cast or brace should be used until radiographic resolution of fragmentation and presence of normal skin temperature (usually needed for 2–4 months)
II (coalescence)	Absorption of debris, sclerosis, fusion of larger fragments	Decreased warmth, decreased swelling, decreased erythema	Total contact casting, prefabricated pneumatic brace, Charcot restraint, orthotic walker or clamshell ankle-foot orthosis
III (reconstruction)	Consolidation of deformity, joint arthrosis, fibrous ankyloses, rounding and smoothing of bone fragments	Absence of warmth, absence of swelling, absence of erythema, stable joint ± fixed deformity	Plantigrade foot: custom inlay shoes with rigid shank and rocker bottom sole
			Nonplantigrade foot or ulceration: debridement, exostectomy, deformity correction or fusion with internal fixation

**Table 2:** Clinicopathological and CT/MRI features of the proposed categories of the Charcot foot.

Category	Clinical symptoms	CT and MRI features	Histopathology
Active stage, grade 0	Mild inflammation (swelling, warmth, pain (?), increased by unprotected walking); no gross deformity	Obligatory: diffuse BMO and STO (Kiuru Grade I–III), no cortical disruption	Lamellar bone with active surface. Remodelling of trabeculae associated with microfractures. Marrow space replaced by loose spindles
		Facultative: subchondral trabecular microfractures (bone bruise); ligament damage	
Active stage, grade 1	Severe inflammation (swelling, warmth, pain (?), increased by unprotected walking); gross deformity, increased by unprotected walking	Obligatory: fracture(s) with cortical disruption, BMO and STO (Kiuru Grade IV)	Increased vascularity of the marrow space, active remodelling of woven bone. Compatible with response to (impaction) fracture. Osteonecrosis. Thickened synovium, fragmented cartilage and subchondral bone, invasion of inflammatory cells and vascular elements
		Facultative: osteoarthritis, cysts, cartilage damage, osteochondrosis, joint effusion, fluid collection, bone erosion/necrosis, bone lysis, debris, bone destruction, bone luxation/subluxation, ligament damage, tenosynovitis, bone dislocation	
Inactive stage, grade 0	No inflammation, no gross deformity	No abnormal imaging or minimal residue BMO; subchondral sclerosis, bone cysts, osteoarthrosis, ligament damage	Sclerosis of bone characterised by broad lamellar trabeculae with collagenous replacement and a low vascularity of the marrow space
Inactive stage, grade 1	No inflammation, persistent gross deformity, ankylosis	Residual BMO, cortical callus (Kiuru Grade IV); joint effusion, subchondral cysts, joint destruction, joint dislocation, fibrosis, osteophyte formation, bone remodelling, cartilage damage, ligament damage, bone sclerosis, ankyloses, pseudoarthrosis	Woven bone, immature and structurally disorganised, fibrosis

BMO, bone marrow oedema; STO, soft tissue oedema.

## Medical treatment

Management should be multidisciplinary around the patient (21). Medical treatment, aside from managing diabetes itself with glycaemic control, involves offloading the lower limb and complete immobilisation of the affected foot using a non-removable total contact cast (22). This is indicated regardless of the location of neuroarthropathy. It has not been proven that offloading with immobilisation is more effective than immobilisation with weight-bearing. The latter would allow for more patient autonomy (23). It is essential to monitor and protect the contralateral foot to prevent bilateral Charcot foot (24, 25). Medical treatment may last 4–6 months, until the clinical or radiological signs disappear (26). Regarding specific pharmacological treatments for Charcot foot, although many molecules have been tested and studied, none have truly proven effective. This includes bone resorption inhibitors such as calcitonin and bisphosphonates. Only immunotherapy with denosumab currently shows effects on bone resorption, but without impact on the duration of remission of the inflammatory phase (27). Bone stimulators such as parathyroid hormone and anti-inflammatory medications such as cortisone have not demonstrated any efficacy (28).

## Surgical treatment

The surgical approach will depend on the anatomical location of Charcot foot.

Before any orthopaedic surgery, a comprehensive arterial assessment (Doppler, CT angiography or contrast-enhanced MRI) should be performed. If vascular intervention is necessary, it must be carried out before any orthopaedic treatment.

Numerous surgical techniques have been described, including percutaneous and arthroscopic approaches, as well as open surgery (29). Similarly, various osteosynthesis methods are used, whether internal (30), external or mixed (31). In percutaneous surgery, procedures such as Achilles tendon lengthening, exostectomies and deformity corrections have been described (32). Arthroscopy is reserved for the ankle and hindfoot. Open surgery is performed for all locations of Charcot foot (33).

Achilles tendon lengthening is primarily indicated to reduce pressure on the forefoot and midfoot, but in peri-talar or even talocrural dislocations, its lengthening is often necessary (34). This may even slow the progression (35) of Charcot foot and reduce the recurrence of ulcers (36). Exostectomies are performed for plantar conflicts, often at a late stage (Eichenholtz stage 3), in the midfoot and hindfoot, for minor deformities. They are currently gaining traction, but the risk remains foot destabilisation. Percutaneous deformity correction is under development and evaluation (37), but early results seem promising (38). There are few studies on arthroscopy in Charcot foot (39). One study, involving arthrodesis with external fixation, reported fewer complications than open surgery but no difference in terms of consolidation and deformity correction.

**Figure 3:**
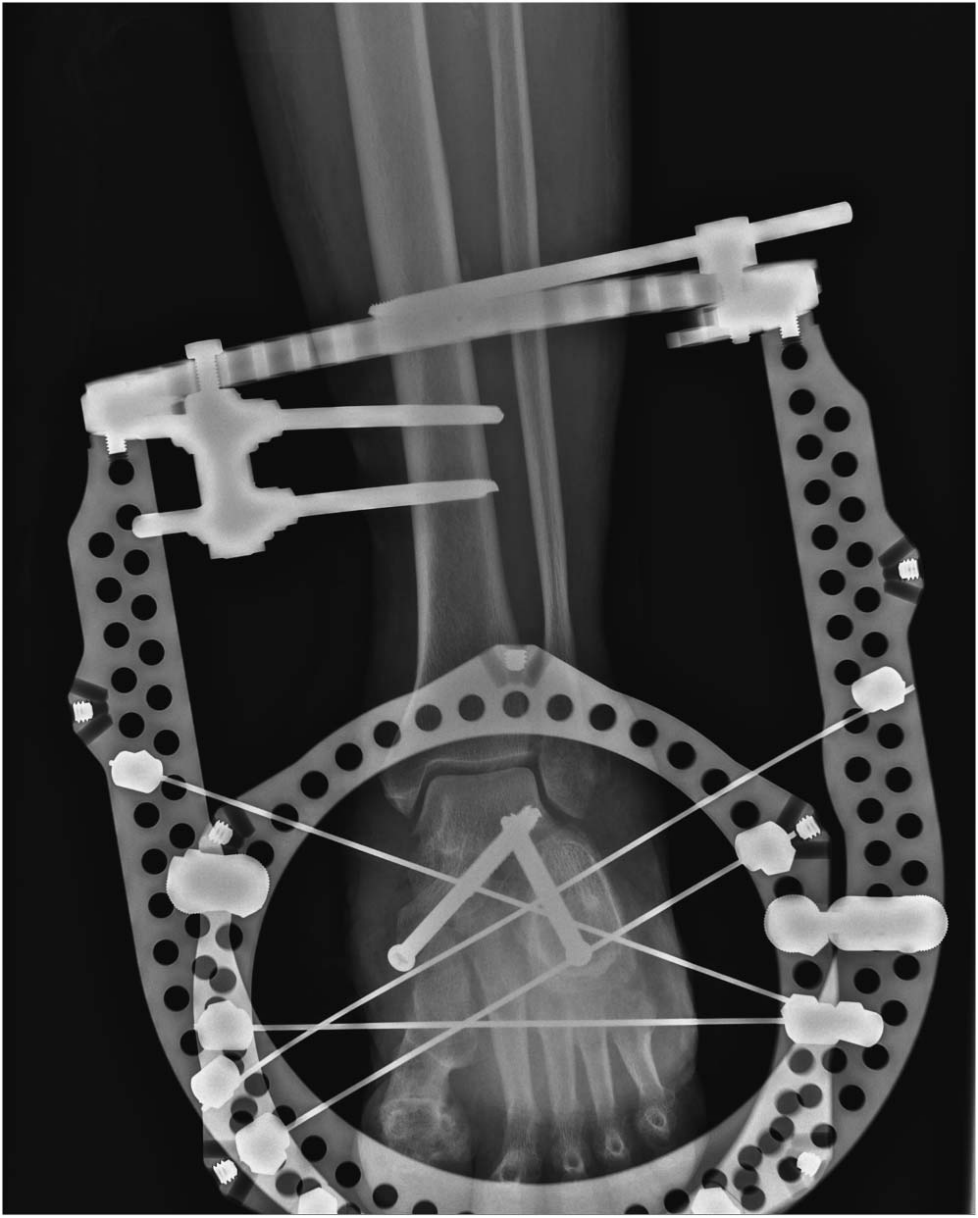
Charcot foot reconstruction.

**Figure 4:**
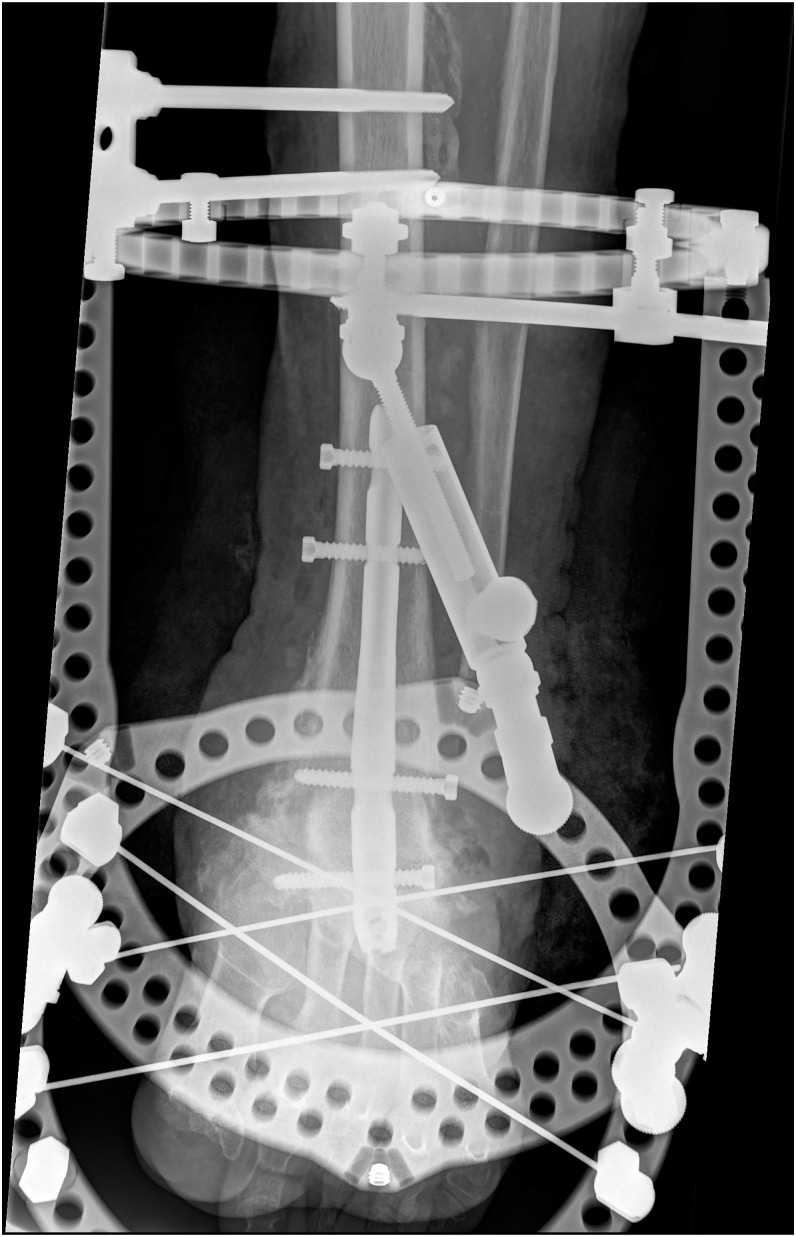
Super construct technique.

Regarding open surgery, it addresses static disorders. The literature describes two major approaches:

- The Charcot foot reconstruction technique, which corrects static disorders while preserving foot bones through various options. This is the most commonly used method with the best outcomes (40) (Fig. 3).
- The ‘super construct’ technique, which immobilises the affected anatomical region as well as adjacent regions, with bone resection if necessary to relieve skin tension while shortening the limb (41) (Fig. 4).

Surgical treatment also includes the debridement of infectious lesions and collection of bacterial samples (42). When Charcot foot is complicated by sepsis, the risk of major lower limb amputation is increased twelvefold. Furthermore, the risk of generalised sepsis is significant, as is the risk of foot destruction. Infection must therefore be managed urgently. Radical debridement should be performed using the ‘Red-Amber-Green’ technique (43), which involves single-stage debridement of all inflamed and infected tissues. As with any osteoarticular infection, broad-spectrum antibiotic therapy should be administered postoperatively according to hospital protocols. Iterative surgical debridements are often required (44). Antibiotic therapy should then be adjusted based on bacterial cultures. In cases of significant tissue loss, negative pressure therapy may sometimes be necessary, although it has not been proven superior to directed healing (45, 46). The management of foot static disorders in such cases will be undertaken at a later stage.

Amputation surgery is also an integral part of Charcot foot treatment, whether as a first-line option or in cases of failed medical or surgical treatment (47). Most cases involve major amputations, defined as above-ankle amputations, which incur the highest costs (48).

Regardless of the surgical treatment, the goal is to achieve a plantigrade, shoe-able foot and to prevent ulcers.

## Indications

Charcot involvement of the ankle and hindfoot corresponds to Sanders’ stages 3 and 4 and Brodsky’s types 2 and 3, representing 10–20% of cases. This is the most common location after the midfoot (49).

When diagnosed early, medical treatment with offloading and non-removable immobilisation has proven effective in reducing the 1-year amputation rate and achieving Charcot remission (normalisation of foot temperature) (50). If, despite well-conducted medical treatment, or if the foot is already deformed at diagnosis, surgery is indicated. Early management of deformities in Eichenholtz stages 0–2, before the appearance of wounds or even deformities, has shown better outcomes (51). At stage 3, surgery is indicated for feet that are minimally or not deformed but remain painful.

For this location, the most commonly used surgical techniques are ankle arthrodesis and tibio-talo-calcaneal arthrodesis (49). Intramedullary nailing, internal osteosynthesis (large-diameter screws, plates) and external fixation (peri-articular fixator) are employed, sometimes in combination. Intramedullary nailing offers the advantage of allowing earlier weight-bearing and stabilising axial and rotational stresses more effectively than external fixation (52). The surgical techniques remain the same regardless of the Eichenholtz stage.

**Figure 5:**
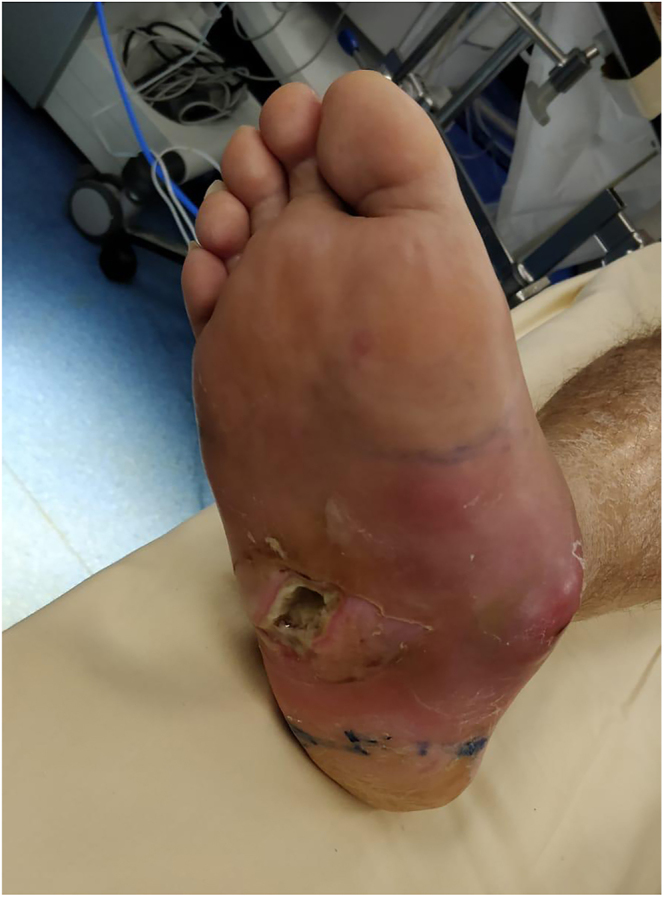
Diabetic foot attack.

**Figure 6:**
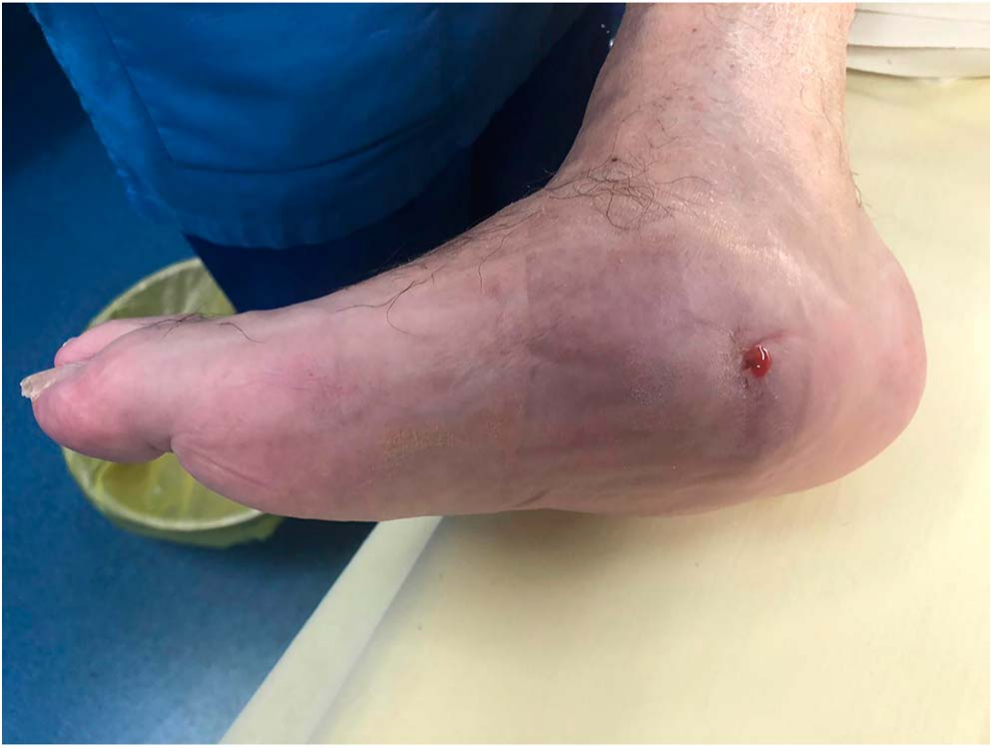
Diabetic foot attack after debridement.

**Figure 7:**
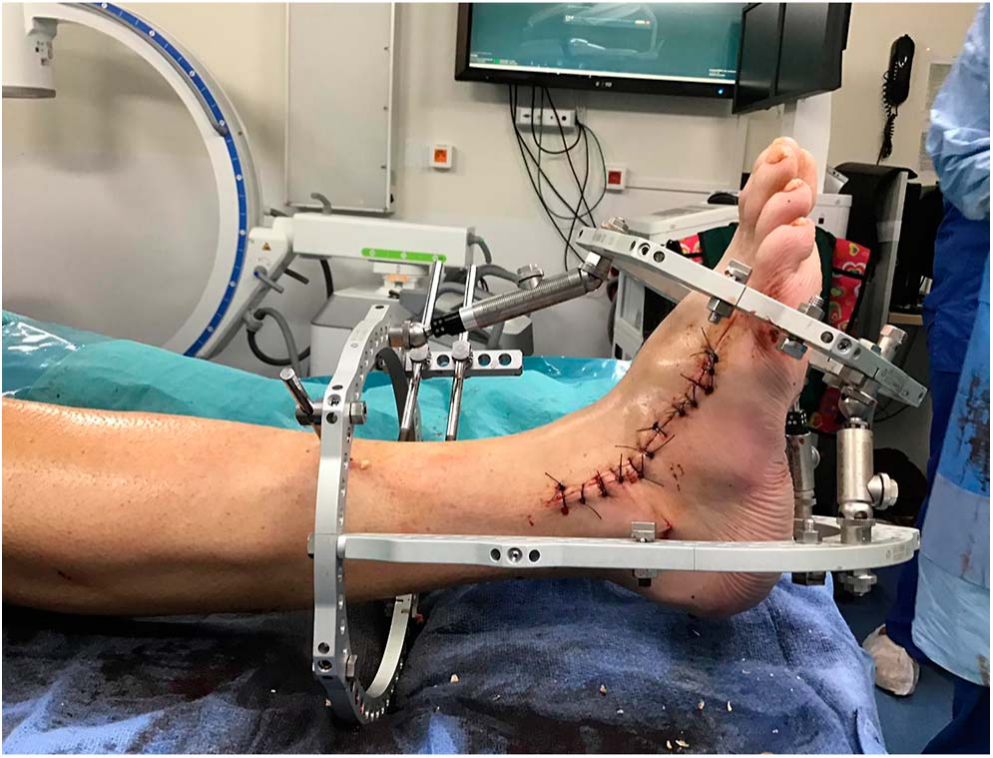
Second stage with morphostatic correction.

The surgical correction of Charcot foot is combined with osteoarticular infection treatment (53). A single-stage surgical approach is performed in cases of chronic sepsis. In cases of acute sepsis, such as ‘diabetic foot attack’ (54), treatment is carried out in two stages: an initial debridement to stabilise the foot (Figs 5 and 6), followed by a second-stage correction of morphostatic disorders (Fig. 7) (55).

## Complications

Regardless of the surgical technique used for Charcot foot treatment in the hindfoot and ankle, complications are frequent (8, 56).

The most common complications of internal osteosynthesis are uncontrolled infection, hardware failure and non-union (57). The use of local bone grafting may reduce the incidence of non-union (58). For external fixation, pin-site infections are the most common complication (59).

A systematic review by Cossins *et al.* on tibio-calcaneal arthrodesis reported an overall complication rate of 69.8% (60). External fixators exhibited the highest complication rates, followed by internal osteosynthesis with screws/plates, and finally, intramedullary nailing, which required no surgical revision. However, external fixation is mainly used in cases of skin ulceration.

In Ha *et al.*’s study, the overall complication rate was 36%, regardless of location. The mixed fixation method had the highest complication rate at 70%. In this study, the amputation rate with foot reconstruction was 5%, but the follow-up period was short.

Despite the high complication rate, surgical management improves patients’ quality of life (61, 62, 63) and reduces mortality rates (64, 65, 66).

## Conclusion

Charcot foot management is evolving rapidly, with an increasing volume of the surgical literature in recent years, although most studies have a low level of evidence.

Management must be multidisciplinary, and diagnosis should be made as early as possible for optimal treatment.

Surgical treatment for hindfoot and ankle Charcot is becoming more common to improve patient quality of life and reduce mortality. Early intervention is increasingly performed as soon as foot deformity occurs, regardless of Eichenholtz stage.

Moreover, surgery is becoming more aggressive, particularly with the ‘super construct’ technique, which appears to yield the best results. Mixed osteosynthesis, combining intramedullary nailing of the hindfoot and peri-articular fixation for overall foot stabilisation, seems to be the best approach despite its high complication rate.

In cases of severe infection, a two-stage surgical approach should be undertaken.

## ICMJE Statement of Interest

The author declares that there is no conflict of interest that could be perceived as prejudicing the impartiality of the research reported.

## Funding Statement

This research did not receive any specific grant from any funding agency in the public, commercial or not-for-profit sector.
